# Targeted temperature management in acute liver failure: A systematic review

**DOI:** 10.1111/nicc.12524

**Published:** 2020-06-29

**Authors:** Juliette Ribaud, Siobhan McLernon, Georg Auzinger

**Affiliations:** ^1^ Intensive Care Unit London Bridge Hospital London UK; ^2^ School of Health and Social Care London South Bank University London UK; ^3^ Liver Intensive Treatment Unit, Institute of Liver Studies King's College Hospital London UK

**Keywords:** acute liver failure, coagulopathy, intracranial hypertension, targeted temperature management, therapeutic hypothermia

## Abstract

**Background:**

Targeted temperature management is the modern term for therapeutic hypothermia, where cooling is induced by intensive care clinicians to achieve body temperatures below 36°C. Its use in acute liver failure to improve refractory intracranial hypertension and patient outcomes is not supported by strong quality evidence.

**Aim:**

This systematic review aims to determine if targeted temperature management improves patient outcome as opposed to normothermia in acute liver failure.

**Methods:**

A computerized and systematic search of six academic and medical databases was conducted using the following keywords: “acute liver failure", “fulminant hepatic injury", “targeted temperature management", “therapeutic hypothermia", and “cooling". Broad criteria were applied to include all types of primary observational studies, from case reports to randomized controlled trials. Standardized tools were used throughout to critically appraise and extract data.

**Findings:**

Nine studies published between 1999 and 2016 were included. Early observational studies suggest a benefit of targeted temperature management in the treatment of refractory intracranial hypertension and in survival. More recent controlled studies do not show such a benefit in the prevention of intracranial hypertension. All studies revealed that the incidence of coagulopathy is not higher in patients treated with targeted temperature management. There remains some uncertainty regarding the increased risk of infection and dysrhythmias. Heterogeneity was found between study types, design, sample sizes, and quality.

**Conclusion:**

Although it does not significantly improve survival, targeted temperature management is efficient in treating episodes of intracranial hypertension and stabilizing an unstable critical care patient without increasing the risk of bleeding. It does not, however, prevent intracranial hypertension. Data heterogeneity may explain the contradictory findings.

**Relevance to Clinical Practice:**

Controlled studies are needed to elucidate the true clinical benefit of targeted temperature management in improving patient outcome.

## 1 BACKGROUND

### 1.1 Acute liver failure and intracranial hypertension

Acute liver failure (ALF) is defined as severe hepatocellular dysfunction,^1^ determined by the presence of coagulopathy (defined by an international normalized ratio ≥ 1.5), jaundice, and evidence of hepatic encephalopathy (HE) without previous history of liver disease. Its most common aetiology in developed countries is paracetamol (APAP) overdose (57% of ALF cases).^2^

Intracranial hypertension (ICH) is defined as an elevated intracranial pressure (ICP) ≥ 25 mmHg.^3^ In ALF patients, ICH is the result of the accumulation of glutamine (synthetized from ammonia in the brain, normally converted into urea by hepatocytes) in the astrocytes, causing swelling and subsequent cerebral oedema. With the disappearance of autoregulation (the ability of the brain to conserve the same blood flow despite changes in perfusion pressures), the inflammatory process increases cerebral blood flow (CBF) and leads to hyperaemia, which in turn raises the ICP.^4^ The main risk of ICH, after cerebral ischaemia, is brainstem death.^5^

The first line of treatment for elevated ICP is a combination of neuroprotective strategies, including deep sedation and analgesia, normo‐ventilation, metabolic and fluid management, haemofiltration, and use of vasopressors to maintain adequate cerebral perfusion pressure (CPP).^6^ The ultimate curative option for ALF is liver transplantation (OLT), although in some cases, spontaneous regeneration is possible if the patient is managed conservatively in intensive care units (ICUs).

### 1.2 Targeted temperature management

Historically, therapeutic hypothermia (TH) has been used to protect the brains of cardiac arrest survivors from secondary injury.^7^, ^8^ The mechanism, although still unclear despite decades of use, is thought to result in a decrease in oxygen consumption by slowing systemic and cerebral metabolism and stabilizing CBF; cooling also has notable anti‐inflammatory properties and seems to interrupt pathways leading to apoptosis (programmed cell death), thus contributing to neuroprotection.^9^

The use of TH for adult brain injuries was rediscovered in the 1990s in a number of clinical trials, when moderate hypothermia (33°C‐35°C) appeared to show equal effects in relation to neuroprotection compared with deep cooling (< 33°C) but without the associated complications.^10^, ^11^ TH also directly leads to ammonia reduction, thus preventing secondary brain swelling in the case of ALF.^12^ Targeted temperature management (TTM) is the newest terminology for TH; the term TTM will be used in the remainder of this review.^13^

The target can be achieved by two main methods: external surface (ice blankets, cooling pads) or endovascular cooling (balloons inflated with cold water inserted in large vessels).

TTM demonstrates good outcomes in animal studies^12^ but still lacks evidence in humans; its use in ALF remains poorly supported by strong evidence, which was the main rationale to conduct this review. Dmello et al. published a systematic review on the use of moderate hypothermia in ALF in 2010^14^; this review also aims to update their results with the latest data available.

Liver failure is defined by the presence of profound coagulopathy (because of the inability of the liver to produce coagulation factors). Because of the effect TTM has on haemostasis, an increased incidence of bleeding complications could potentially be an issue in ALF.^12^, ^15^ Secondly, because of the central role the liver plays in immune function through Kupffer cells and as the largest phagocytic organ in the body, as well as immune regulator, ALF itself impairs the immune function and, in combination with TTM, may increase the risk of infectious complications.

## 2 AIMS AND OBJECTIVES

The aim of this review is to determine if TTM improves patient outcomes in ALF.

The following objectives aim to establish if TTM:

- Improves survival,
- Reduces refractory intracranial hypertension,
- Prevents ICH, and
- If its benefits outweigh the associated complications.

## 3 METHODOLOGY

### 3.1 Search strategy

No date range has been set in order to include more studies. The first case report on ALF is from 1999,^16^ quickly followed by the first cohort study.^17^ All study types are included in this review, except for qualitative studies, not relevant to the research question.

The bibliographies of primary and secondary studies were scanned. Authors were contacted through ResearchGate or in person.

### 3.2 Databases and keywords

Four academic databases were separately searched: Medline, CINAHL, Academic Search Complete, and PubMed. Alternative databases, such as Google Scholar and Science Direct, were also browsed to complete the search using a modified strategy without Boolean operators.

The following keywords were used: “acute liver failure", “fulminant hepatic injury", “targeted temperature management", “therapeutic hypothermia", and “cooling". No outcome keywords were used in the search; inclusion was conducted manually.

### 3.3 Exclusion criteria

To broaden the search to a maximum in the absence of a satisfactory number of good‐quality results, only minimal restrictions were applied to the search: paediatric and animal studies, the use of TTM for other aetiologies, studies not in English or French, secondary studies, editorials, and books were excluded.

### 3.4 Sifting and sorting process

**FIGURE 1:**
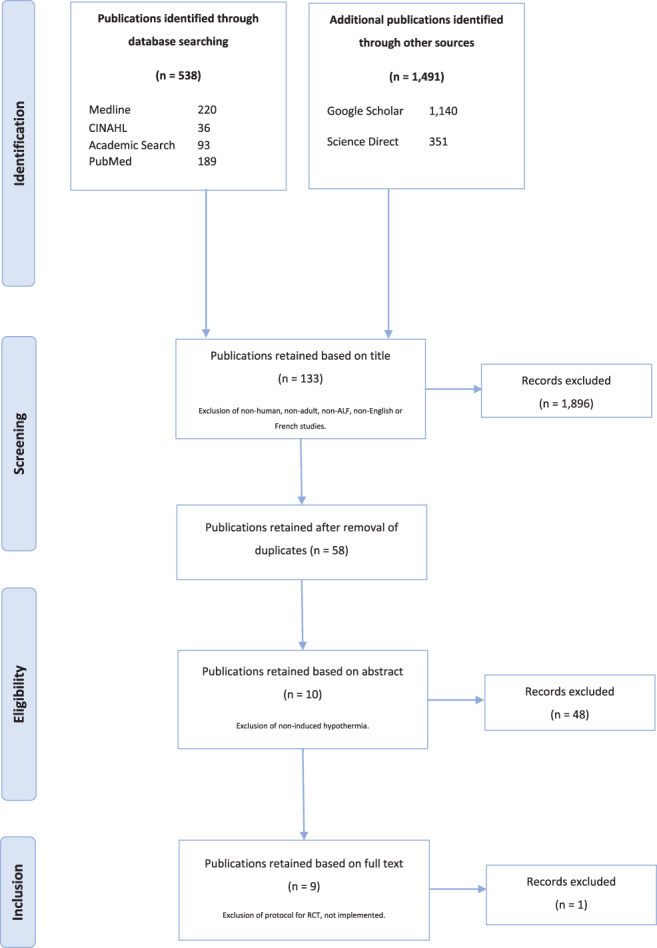
Modified PRISMA chart representing the sifting process conducted in April 2019

Following the first database search in April 2019, 2,029 articles were found across academic and additional databases (Figure 1); 1,896 articles were discarded according to the above criteria as per their title. A second sifting process removed another 48 based on their abstract, including a study where hypothermia was not induced by clinicians.^18^ Finally, after full‐text review, nine studies were included in the review. An article preparing for the implementation of a randomized controlled trial (RCT) published in 2008 was excluded as it was not converted into a formal trial and therefore presented no exploitable data.^19^

### 3.5 Data extraction, critical appraisal, and narrative synthesis

The data have been extracted using a template for quantitative studies from Noyes and Levin^20^ and adapted for this review.

The Critical Appraisal Skill Program (CASP) tool was used for the quality appraisal process for RCTs and cohort studies.^21^ Case reports were appraised using a tool developed by Roever and Reis.^22^ Guidance from the Cochrane Group was also consulted to synthesize the results.^23^

## 4 RESULTS

### 4.1 Preliminary synthesis

Among the nine included studies (Table 1), four originated from the same team, Jalan and colleagues, and were published within 5 years (1999‐2004). Two patients from their first paper^17^ were re‐sampled in the next trial^24^; the authors did not specify which patients were studied again.

**TABLE 1:** Study designs, aims, findings, and limitations of reviewed studies

Authors, date	Study design	Methods	Aims	Findings	Limitations
Bernal et al, 2016	Sample: 43 Type: Multi‐centre RCT	Settings: 3 ICUs (UK and DK) Population: ALF pre‐OLT; median age 38; aetiology APAP 70% Intervention: ICH prophylaxis; TTM method: surface ± CRRT, target 34°C for 72 h Outcomes: ICP; mortality; adverse events National randomization in 2 groups	to investigate if maintenance of MH prevented development of ICH in ALF patients at high risk of the complication	No benefit in mortality for use of TH for prophylaxis: overall mortality: 41% in TH and 46% in ctrl (*P* = .75) Non‐OLT mortality: 70% in TH and 57% in ctrl (*P* = .52) OLT mortality: 0% in TH and 33% in ctrl (*P* = .09) No benefit to prevent ICH: 19/43 (10 in TH; 9 in ctrl) developed ICH Mean ICP & CPP similar in both groups Occurrence of side effects similar in both groups (35% vs 42%; *P* = .83) Coagulopathy 6% vs 25% (*P* = .4) Haemorrhage 0% vs 12% (*P* = .38) Sepsis 18% vs 25% (*P* = .85) Cardiac events 12% vs 4% (*P* = .73)	Medium sample, unequal group sizes ICH treated differently Trial stopped before projected end because of increased incidence of ICH and futility Methods of cooling not detailed
Holena et al, 2012	Sample: 1 Type: Case report	Setting: ICU (US) Population: ALF post‐OLT; age 23; aetiology APAP Intervention: ICH treatment; TTM method: surface, target 33°C for 60 h Outcomes: ICP; survival; neurological complications Single case	to investigate the effect of TH for intractable ICH after liver transplantation	Patient survived OLT without neurological deficit after day 30 post‐op on home discharge Significant decrease in ICP after TH initiation, then maintained stable <20 mm Hg for the TH duration; no further ICP spikes observed No evidence of adverse effects	Lack of referenced definition Could have benefitted from detailed data (table) Case report, weak evidence Short‐term follow up (30 d) TH initiated 2 d post‐OLT; efficacy could be attributed to the good graft function
Jacob et al, 2009	Sample: 1 Type: Case report	Setting: ICU (US) Population: ALF (*not listed for OLT*); age 27; aetiology APAP Intervention: ICH treatment; TTM method: surface, target 32.8°C for ~120 h Outcomes: ICP; survival; neurological complications; ammonia Single case	to review evidence‐based treatment options in patients with cerebral oedema complicating FHF and discuss the potential applications of hypothermia	Patient survived without OLT without neurological deficit ICP decreased to <20 mm Hg after 2 h of TH and then remained stable for the next 4 days No evidence of adverse effects	Unclear graph, difficult to read. Unavailable conversion of ammonia as brut data. Case report, weak evidence
Jalan et al, 1999	Sample: 7 Type: Prospective single‐centre cohort study	Setting: 1 ICU (UK) Population: ALF pre‐OLT; median age 29; aetiology APAP 86% Intervention: ICH treatment; TTM method: surface, target 32°C to 33°C for ~12 h Outcomes: survival; CBF; metabolic indices 2 groups: listed for OLT (*n* = 4) vs non‐listed (*n* = 3)	to assess the efficacy and safety of MH in the management of patients with ALF	Uncertain benefit in mortality when transplanted: all 4 transplanted patients survived OLT (1 died 3 days later from herniation) all 3 not‐listed patients died after rewarming (rapid rewarming) Benefit in lowering ICP and CBF: drop in ICP in all patients within 1 h of cooling CBF: improved after 4 h of cooling (*P* < .05) No evidence of significant bleeding or infection (1 patient developed infection after OLT)	Group allocation based on psychological/historical reasons rather than clinical Only one group is treated with OLT and one without: questionable choice for survival and efficacy measurement as primary outcome Rewarming protocol (over 2 h) too abrupt for survival in non‐OLT group? Limited follow up Cohort study, not randomized, small sample, single centre; same team and same centre as next 3 studies
Jalan et al, 2001	Sample: 9 Type: Prospective single‐centre cohort study	Setting: 1 ICU (UK) Population: ALF; median age 32; aetiology APAP 78% Intervention: ICH treatment; TTM method: surface, target 32°C for ~4 h Outcomes: CBF; CO_2_ reactivity; ICP; metabolic indices	to assess if MH is reducing ICP by restoring CBF autoregulation	No data on mortality nor complications. 100% patients showed CBF autoregulation restored 4 h after cooling (*P* < .04) 5 patients (measured) responded to the increase of pCO_2_ after cooling where they did not respond prior (*P* < .05)	Recruitment not detailed; 2 patients resampled from previous study Cohort study, not randomized, small sample, single centre; same team and same centre Not all patients' CO_2_ was measured No follow up
Jalan et al, 2003	Sample: 16 Type: Prospective single‐centre cohort study	Setting: 1 ICU (UK) Population: ALF peri‐ & post‐OLT; median age 27; aetiology APAP 86% Intervention: ICH treatment; TTM method: surface ± CRRT, target 33.5°C for ~36 h Outcomes: ICP; survival; CBF; metabolic indices 3 groups: 2 normothermic (*n* = 6 & 5) vs TTM (*n* = 5)	to test the hypothesis that MH would prevent the increases in ICP during OLT	Similar mortality in all three groups: 1 patient died in each group from MOF None of the patients demonstrated neurological defects after OLT ICP stability improved at lower temperatures (group 3), with large fluctuations of ICP and CBF in non‐TH groups (1 & 2) Complications: No significant increase in risk of infection No increase in coagulation factors, blood products, or fluids transfused during transplant	Cohort study, not randomized, small sample, single centre; same team and same centre Missing details in definitions used (ALF, MH), intervention (method, duration), implementation, choice of outcomes. Measurement of interleukin not discussed in introduction and seems like an afterthought. Pertinence is therefore questionable, although one can see the point. But as it was not measured for all patients, it could have been integrated in another study instead
Jalan et al, 2004	Sample: 14 Type: Prospective single‐centre cohort study	Setting: 1 ICU (UK) Population: ALF pre‐OLT; median age 24; aetiology APAP 93% Intervention: ICH treatment; TTM method: surface, target 33°C for ~32 h Outcomes: survival; ICP; CBF; adverse effects; metabolic indices No group	to evaluate the clinical effects and pathophysiologic basis of hypothermia in patients with ALF and ICH that are unresponsive to standard medical therapy to be used as a bridge to transplantation	Overall survival post‐OLT is 77% 13/14 included patients were transplanted; 1 died from brain herniation 3 patients died within 3 mo from MOF (sepsis?) and PGNF Significant decrease in ICP after 4 h of cooling (*P* < .001) Significant CBF lowering after 4 h (*P* < .001) Sustained improvement during treatment No evidence of increased bleeding during MH but potential increased risk of infection: no bleeding during insertion of ICP bolt nor other procedure while having MH; “regular” amount of blood products administered during transplant 9/13 developed infection	Graphs difficult to read and interpret Cohort study, not randomized, small sample, single centre
Karvellas et al, 2015	Sample: 1232 Type: Retrospective multi‐centre cohort study	Settings: 28 ICUs (US & CA) Population: ALF pre‐OLT; median age 38; aetiology APAP 49% Intervention: ICH prophylaxis; TTM method: surface, target 33°C to 35°C for 24 to 72 h Outcomes: survival; adverse effects 2 groups: TH (*n* = 97) vs control (*n* = 1135)	to determine the impact of TH on 21‐day survival and complications in ALF patients at high risk for cerebral oedema	Minimal significance on overall mortality: Spontaneous survival better in TH group (45% vs 39%; *P* = .24) without difference of aetiology Mortality because of solely neuro complications similar in TH and control group Positive effect found between age and TH for spontaneous survival in APAP group (*P* = .024) Complications: Infections: VAP and sepsis rates lower in TH groups (*P* = 61) Coagulopathies: GI bleed rate similar in both groups but weak significance (*P* = .95) Arrhythmias: TH patients more likely to have arrhythmias than control (*P* = .03) Duration of TH treatment has no impact on complications occurrence	Retrospective analysis (potential selection bias), no randomization Unequal group sizes Local standards of care, protocols, and listing criteria different in each centre; no uniformity in timing or criteria for use of TH Short timeframe for follow up
Roberts and Manas, 1999	Sample: 1 Type: Case report	Setting: ICU (UK) Population: ALF peri‐ & post‐OLT; age 20; aetiology APAP Intervention: ICH treatment; TTM method: surface, target 32°C for 24 h Outcomes: ICP; survival; neurological complications Single case	to determine if hypothermia in the management of raised ICP before and after liver transplantation has beneficial outcome	Patient survived OLT without evidence of neurological deficit on 14‐mo follow up ICP decreased by 50% during OLT but raised again to 55 mm Hg after 48 h post‐op (on discontinuation of TH) No data on adverse effects	Confounding factors OLT heavily impacts the assessment of TH efficacy Case report, weak evidence

*Note*: ALF, acute liver failure; APAP, paracetamol overdose; CA, Canada; CBF, cerebral blood flow; CO_2_, carbon dioxide; CRRT, continuous renal replacement therapy; DK, Denmark; ICH, intracranial hypertension; ICP, intracranial pressure; ICU, intensive care unit; MH, moderate hypothermia; OLT: orthotopic liver transplantation; RCT, randomized controlled trial; TH, therapeutic hypothermia; TTM, targeted temperature management; UK, United Kingdom; US, United States.

Most of the literature in this review is dated, with only three studies published after 2010; the median year of publication is 2004. In the context of fast‐paced technological and medical progresses, valid findings from 10 years ago might not be relevant today; five studies fall in that time window.^4^, ^16^, ^17^, ^24^, ^25^ However, the two latest publications^26^, ^27^ are also the strongest in terms of methodological quality and sample size and are both multicentred studies. The last study from 2016 is the only RCT included in this review. The three case reports^16^, ^28^, ^29^ only discuss one patient each and constitute the weakest type of observational studies available. The five cohort series are diverse in terms of sample size (from 7 to 1,232 patients), design, and quality.

### 4.2 Quality appraisal

The quality of the reviewed studies is disparate. The first two cohorts from Jalan^17^, ^24^ demonstrate flaws in the study design, failing to identify confounding factors and displaying insufficient follow up. The last three cohorts^4^, ^25^, ^26^ show some improvement in reliability and dependability. Bernal's RCT^27^ displays rigorous methodology. Aside from the reporting bias inherent in case studies (reporting only successful cases), the case reports met all criteria on scrutiny.

All studies address a focused issue, clearly identifiable from the title, and all are applicable to the local population as they distinctly recruit ALF patients matching previous or current consensus definitions. The demographics also match ALF subjects and facilitates the transferability. The length of follow up is the weakest point across all the included studies. It is absent in one study^24^ and rarely extends beyond 30 days post‐discharge in the others.

### 4.3 Findings

#### 4.3.1 Survival

In the studies investigating the efficiency of TTM to treat ICH, survival is high. All three case studies report 100% survival without neurological complications at a minimum of 30 days post‐discharge; each one describes a different indication for hypothermia: peri‐OLT,^16^ post‐OLT^28^ and not listed for transplant.^29^

The cohort studies display relatively high survival rates at the end of the follow‐up period; however, most patients who survived were transplanted either before or after TTM implementation.^4^, ^17^, ^25^ Because of the transplant censoring, it is unclear what—if any—effect the intervention had on outcomes.

In the latest studies, authors investigated the use of TTM to *prevent* ICH. The survival rates in the large retrospective cohort^26^ and the only prospective RCT^27^ are significantly different from the previous observational and largely retrospective case studies and series on the treatment of established ICH.

In Karvellas' cohort, the retrospective analysis of 15 years' worth of data showed that TTM does not significantly improve either overall or transplant‐free survival, although the spontaneous survival (without OLT) is higher in the hypothermia group without reaching statistical significance (45% vs 39%, *P* = .24 *P* = .024); the same correlation was not found in non‐APAP aetiologies. In Bernal's RCT, there was no survival benefit for TTM compared with control patients, nor was there a decrease in the incidence of intracranial pressure surges (59% vs 54%, *P* = .75).^27^ The only noteworthy finding was the 100% survival of transplanted patients in the hypothermia group, as opposed to 77% who survived in the control group (*P* = .09). It is therefore difficult to correlate the efficacy of TTM with improved survival in the hypothermic group.

Overall, the survival rates between TTM and normothermic groups are similar (63% vs 60%, respectively). A comparison of studies showed very heterogeneous sample sizes and no outcome benefit for the intervention groups, with a mean *P* value of .7.^25^, ^26^, ^27^

#### 4.3.2 Intracranial pressure and neurological parameters

Most studies showed a significant and early—within 1 hour—decrease in ICP when TTM was implemented. The magnitude of reduction (ΔICP) is similar notwithstanding the duration of hypothermia (average of −20 mmHg); most patients saw a 2‐fold improvement and successful normalization of ICP below 20 mmHg from an average of 40 mmHg prior to cooling, but this was restricted to case reports and small sample cohorts.

**FIGURE 2:**
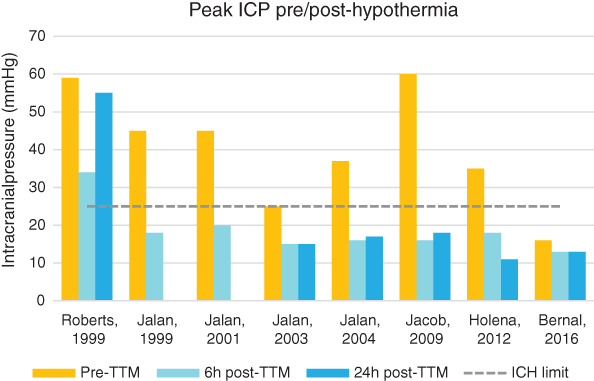
Histograms of mean peak intracranial pressure (ICP) differences pre‐ and post‐targeted temperature management (TTM) by year of publication

On the contrary, Bernal—while investigating the prevention of ICH (with ICPs lower than 15 mmHg)—noticed that hypothermic patients tended to have higher ICP than normothermic patients during ICH episodes.^27^ Of 43 patients, 19 eventually developed ICH—10 in the TH group and 9 in the control group (*P* = .43), for an overall occurrence of 35% and 27%, respectively (Figure 2). Although the targeted temperature difference between TTM (34°C) and control group patients was achieved, temperature rise above 36°C was still avoided in controls.

Jalan^24^ observed restoration of CBF autoregulation shortly after cooling patients to 32°C (*P* < .04), as well as the re‐establishment of CO_2_ reactivity (the ability of cerebral vessels to constrict and dilate depending on carbon dioxide concentration) in 71% of patients (*P* < .05). In 1999 and 2004, Jalan's team calculated CBF and noticed a significant improvement with a mean ΔCBF of −46 mL/100 g/min (*P* < .05).^4^, ^17^ The same trends were measured during transplantation in 2003^25^: there were large variations in CBF in normothermic groups, depending on the various phases of liver transplant surgery. The TTM group maintained relatively stable CBF values between 45 and 50 mL/100 g/min throughout.

#### 4.3.3 Adverse events

Overall, studies confirm that, despite a generally increased bleeding risk in ALF, induced hypothermia does not increase the risk of bleeding independently^17^, ^26^, ^27^, ^28^, ^29^; the amount of blood products transfused was also not increased in hypothermic patients.^4^, ^25^ In a few cases, the incidence of haemorrhage or thrombocytopaenia was lower than in control groups (*P* = .4).^27^

Two studies mention a higher incidence of arrhythmias: Bernal et al. noticed a non‐significant numerical difference (12% vs 4% in control, *P* = .73) and only in cases where the patient's temperature decreased to below the target of 34°C.^27^ Karvellas found that 38% of hypothermic patients suffered from dysrhythmias (*P* = .03) compared with 27% in normothermia patients.^26^

Regarding an increased infection risk, findings are mixed. Sepsis, ventilator‐acquired pneumonia (VAP), or other kinds of infections occur in both hypothermic and control groups, without a clear link between TTM, the degree of cooling, and the duration of treatment. In the RCT, the incidence of sepsis was higher in the control groups compared with TH (18% vs 25%, *P* = .85).^27^ In Karvellas' cohort, the overall incidence of VAP was not different between groups (20% vs 23%, *P* = .55); in a subgroup analysis of patients whose temperature decreased to below 33°C, there was a higher risk of developing VAP (35% vs 15%, *P* = .03).^26^

Authors generally agree that clinically significant side effects because of TTM are rare and not more common than in control patients.

#### 4.3.4 Other significant findings

TTM improved haemodynamic stability in several trials.^4^, ^17^, ^24^, ^25^, ^27^, ^28^ Partially linked to the restoration of autoregulation and peripheral vasoconstriction, blood pressure increases at lower temperatures, thus reducing vasopressors requirements. Holena postulates that TTM creates stability where other treatments were not successful,^28^ and Jalan notes that hypothermia is similarly helpful to maintain stability during transplant, especially during graft reperfusion, a critical phase of OLT that usually generates profound cardiovascular instability.^25^

## 5 DISCUSSION

### 5.1 Outcomes

No significant survival benefit or improvement in neurological outcome has been found for TTM in ALF. Findings from more recent and higher‐quality investigations^26^, ^27^ contradict earlier studies,^4^, ^16^, ^17^, ^24^, ^25^, ^28^, ^29^ although those focused more on the treatment of ICH rather than preventing it. Bias through liver transplantation, the only curative intervention for ALF, regardless of aetiology, age, gender, and hypothermia status, makes it even more difficult to evaluate if TTM can independently improve survival and long‐term outcomes.

As it is the case in the ALF population in general, trials that can isolate hypothermia from any other intervention are nearly impossible to design. As summarized by Karvellas,^26^ TTM does not seem to result in an outcome benefit in ALF patients, but it does not seem to cause harm either.

An interesting perspective was raised by Jalan^4^ as to whether TTM could be used as a bridge to transplantation, thus indirectly helping survival. Of the patients studied in those trials, 93% were successfully bridged to OLT, with a subsequent 71% post‐transplant long‐term survival in 2004. This is consistent with previous studies^17^ where survival after OLT was 75%, and 0% when patients fulfilled poor prognostic criteria and were not transplanted. However, this should be readjusted according to chronological context: an emerging trend during the last 10 years favours non‐transplant management with paracetamol aetiology where significant improvements in spontaneous survival have been reported across Europe and the United States.^30^

Furthermore, when considering the time effect in ALF management, the incidence of ICH‐related deaths has significantly decreased over the last two decades because of progresses in critical care management of these patients. ALF patients are now more readily referred to tertiary specialist centres and accordingly transferred earlier; they are more aggressively managed in critical care despite a trend towards the reduction of invasive monitoring of the ICP; and the use of extra‐corporeal blood‐purification techniques are now common in specialist ICUs. Deaths attributed to neurological causes has decreased significantly in developed countries and is expected to decrease further in the coming years.^30^

Nonetheless, TTM clearly seems to improve ICH, although the exact mechanisms of action have not been fully elucidated. Several hypotheses arose over the last few decades, including the decrease of cerebral metabolic rate for oxygen, improvements in cerebro‐ and cardiovascular haemodynamics, and reduction in the production of inflammatory mediators.^28^ Jalan et al.^25^ also noticed that the restoration of CBF autoregulation immediately after cooling is specific to ALF; the authors note that it can take days to reappear in other conditions such as traumatic brain injury (TBI) or meningitis.

Regarding the prevention of ICH, Bernal^27^ confirms that hypothermia does not significantly improve outcomes, nor does it prevent ICH episodes. Those findings are unexpected and contradict previous findings, as well as animal studies performed on rats.^12^ The reason hypothermia would treat but not prevent ICH is yet to be explained. Avoidance of hyperthermia may be ultimately as effective as induced hypothermia and associated side effects.

The benefits of TTM on cardiovascular stability requirements are consistent in the results of this review. Achieving cardiovascular stability is one of the core management goals in critically ill patients and is often a challenging task for nurses and clinicians. This raises the question of whether TTM, even in the absence of benefits for survival or prevention of ICH, could potentially be used “solely” to stabilize otherwise haemodynamically unstable patients.

The methodological rigour of Bernal's RCT^27^ and Karvellas' cohort^26^ outperforms the previous observational studies in design, sample size, and methodological quality and hence are scientifically more valid.

### 5.2 TTM methods and process

The depth of hypothermia achieved has evolved over the last two decades, with mean targets increasing by 2°C in the latest trials compared with 20 years ago (from means of 32°C to 34°C in the latest studies). Given the data heterogeneity, it is difficult to find a correlation between the evolution of target temperatures and the efficacy of TTM on ICP reduction. The same goes for the evolution of survival rates depending on the target temperatures. However, aside from a mildly higher incidence of infection at lower temperatures, the depth of cooling does not increase the occurrence of adverse events.

The duration of treatment in the evaluated studies varies from a few hours^17^ to 5 days,^29^ with a mean duration of 46 hours over all studies. All teams used surface cooling, and only in a few cases was this combined with the continuous renal replacement therapy (CRRT) circuit,^25^, ^27^ and none used endovascular devices—a widespread cooling method in post‐cardiac arrest or neurological trauma cases.^31^, ^32^

A few studies highlighted the importance of the rewarming phase.^16^, ^17^ When patients are rewarmed too quickly, it can lead to dangerous rebound ICP surges, which can be fatal. The rewarming from 32°C to normothermia occurred over 2 hours (+2.5°C/h), whereas it is now recommended to rewarm at a rate of 0.1°C to 0.5°C per hour.^8^

### 5.3 Summary of findings

The heterogeneity of the included studies, both in design and findings, prevents a formal and high‐quality evidence‐based answer to the research question and this review's objectives.

Overall, TTM seems to improve short‐term outcomes such as high ICP and haemodynamic instability. Temperatures between 32°C and 34°C effectively treat refractory ICH but do not prevent the occurrence of such events. TTM does not worsen coagulopathies or increase the haemorrhagic risk. It might mildly increase the chances of dysrhythmias and pulmonary infections; its effect on the incidence of nosocomial sepsis rates is inconclusive.

However, TTM has not shown any major impact on survival. If a few isolated early cases reported a direct relationship between its use and survival or neurological recovery, methodologically stronger studies mitigate those findings and fail to demonstrate clearly any difference between treatment and control groups in terms of survival. Overall, human controlled studies contradict uncontrolled data and animal studies and their appealing pathophysiological concept of TTM as an effective treatment of ICH associated with ALF.^33^

### 5.4 Comparison with previous reviews

In their systematic review, Dmello and colleagues^14^ obtained similar results of ICP (ΔICP = −22 mmHg) and CBF reduction (ΔCBF = −49 mL/100 g/min). The findings of this review come to similar conclusions: mean ΔICP and ΔCBF estimated at −20 mmHg and −46 mL/100 g/min, respectively. The findings discussed in Dmello's review are limited,^14^ but the available results confirm this review's conclusions.

A Cochrane systematic review on the use of TTM for traumatic brain injury published by Lewis et al.^34^ concluded, from low‐quality evidence, that it is currently impossible to determine if the intervention reduces the risk of death or severe disability or increases the risk of respiratory infection. Arrich et al.^35^ published a similar review after cardiopulmonary resuscitation, and they concluded that, although originating from moderate‐quality studies, hypothermia increased the chances of favourable neurological outcome and provided 30% more chances of survival than control groups. Aside from a slightly higher risk of pneumonia, they found no increased risk of adverse events.

The findings of Arrish^35^ diverge slightly from this review's and Lewis' conclusions as the authors are more confident that TTM improves survival and neurological recovery. All reviews agree that TTM does not significantly increase the risk of adverse events, and it can therefore be considered a relatively safe intervention.

### 5.5 Nursing considerations

There is currently no primary research in nursing for the use of TTM in ALF. Available publications in critical care nursing journals, all secondary research, focus on purely neurological indications. The available recommendations are weak and originate from low‐quality evidence.^9^

Different phases of the treatment involve specific monitoring and nursing care. Initiation refers to the beginning of TTM, where the cooling is started by one or several methods. The maintenance starts when the target temperature has been achieved and aims to prevent large variations in temperature because of external interventions. Finally, the rewarming involves the monitoring of haemodynamic parameters to avoid the downfalls that Roberts & Manas and Jalan experienced.^16^, ^17^ The latest devices automatically prevent this from happening but still require attentive nursing monitoring.

The methods of cooling briefly mentioned above also impact nursing care. The choice of surface devices de facto creates an extra layer above and beneath the patient that, in the case of critically ill patients with multiple comorbidities, can directly cause pressure ulcers. The use of cooling/ice packs can also cause cold burns to the unprotected skin.

The use of endovascular devices adds a supplementary central venous access, which needs to be closely monitored by nurses to detect infection or dislodgement. Ultimately, the use of the CRRT machine's temperature control system is, as described by Bernal and colleagues,^27^ the “easiest method of cooling” as it does not involve supplementary tools, but precise target temperature may be difficult to achieve and maintain.^36^

### 5.6 Limitations

The main weakness of this review is the lack of recent studies and the scarceness of evidence available. The inclusion of case reports, as well as cohort studies of relative quality, does ultimately have an impact on the conclusions that can be drawn. Furthermore, the large heterogeneity in sample size, design, and findings also prevents definitive and high‐quality conclusions to the research question. The transplant censoring in the results questions the feasibility of separating any intervention in ALF with the curative option of OLT in measuring outcomes.

### 5.7 Recommendations

This important topic would benefit from a larger multicentre RCT. However, even the methodologically strongest RCT will be unable to control all residual confounding factors fully, especially with the curative intervention of OLT. Nonetheless, further large, multicentred RCTs are essential to strengthen the currently available evidence and advice for uniform protocolized ICU management in ALF. A future trial could isolate specific categories of population, for example, the young population after paracetamol overdose and those not listed for transplantation (with chances of spontaneous recovery). Such a trial could then measure if TTM improves their outcomes, such as survival or hepatic regeneration, without the variable set by transplantation.^33^

Other trials could also aim to answer questions about the intervention parameters, such as what the optimal target temperature is and for which indication (ALF, TBI, post‐resuscitation), or what is the ideal method of cooling that conveys the most benefits (machine and consumables cost, easiness of use) with fewer risks of complications (invasiveness, pressure ulcers).

A nursing observational study could also focus on the challenges of cooling from the nurses' perspective, exploring the care and specific needs during initiation, maintenance, and rewarming phases.

## 6 IMPLICATIONS FOR CLINICAL PRACTICE

This systematic review has presented an up‐to‐date review of the use of TTM in ALF. While it is a relatively common practice to cool patients for neuroprotection in ICU, evidence is scarce in relation to its efficacy in improving patient outcome. Although it does not significantly improve survival, TTM is efficient in treating episodes of ICH and stabilizing an unstable critical care patient without increasing the risk of bleeding. It does not, however, prevent ICH.

## 7 CONCLUSION

This systematic review aimed to assess if the use of TTM improved patient outcome in ALF. A computerized search resulted in the inclusion of nine primary studies of disparate type, sample size, and methodological quality but were all applicable to the ALF population with confirmed ICH or at risk of developing it. Few studies were conducted within the last decade, thus weakening the findings with a conclusion that might not entirely be contemporary in today's critical care settings.

The main outcomes measured were survival, the treatment of refractory ICH, and the prevention of ICH. Finally, the occurrence of adverse events such as coagulopathies, dysrhythmias, and infections was investigated and put into the context of ALF, where bleedings and infections are notoriously more frequent than in the general critically ill population.

The heterogeneity of the studies in type, design, variables, outcomes, and quality; the low significance of results; and the sometimes‐contradictory findings prevent a high‐quality and definite answer to the research question. However, some general lines can be drawn. TTM does not positively affect survival. Nonetheless, it helps to reduce ICP during episodes of ICH, including in situations where the ICP did not respond to conventional medical treatment. Cooling also restores CBF autoregulation, therefore contributing to the neuroprotection of brain cells; it also stabilizes otherwise haemodynamically unstable patients. Paradoxically, TTM does not prevent ICH episodes, therefore not advocating for the use of prophylactic hypothermia before signs of ICH begin to appear.

The occurrence of adverse effects of hypothermia did not outweigh the benefits, making TTM a safe intervention in critically ill patients with ALF. Those patients receiving TTM were not more susceptible to bleeding than normothermic patients. They possibly had a slightly higher rate of dysrhythmias and infections at low temperatures, but none of the authors concluded that those risks were legitimately a contraindication.

The absence of nursing input about TTM, furthermore in ALF population, is a big gap in the evidence‐based practice current medical and paramedical sciences are aiming for. The main limitations of this review concern the lack of recent and high‐quality studies to answer the research question with certitude. More controlled trials are needed to clarify the true clinical benefit of TTM in treating ICH.

## AUTHOR CONTRIBUTIONS

JR: MSc dissertation author, re‐edititing for journal publication; SMcL: data extraction and quality appraisal, grading as dissertation supervisor, review, and support; GA: review of clinical content and phraseology.
